# Editorial: Global excellence in health economics: Asia and Australasia

**DOI:** 10.3389/fpubh.2023.1172632

**Published:** 2023-03-24

**Authors:** Mihajlo Jakovljevic, Hanyu Chang, Narimasa Kumagai

**Affiliations:** ^1^Institute of Advanced Manufacturing Technologies, Peter the Great St. Petersburg Polytechnic University, St. Petersburg, Russia; ^2^Institute of Comparative Economic Studies, Hosei University, Tokyo, Japan; ^3^Department of Global Health Economics and Policy, University of Kragujevac, Kragujevac, Serbia; ^4^First Hospital of Lanzhou University, Lanzhou, China; ^5^Department of Economics, Seinan Gakuin University, Fukuoka, Japan

**Keywords:** global collaboration, health economics, public health, health insurance, pharmaceutical expenditure, developing countries, medical care

There has been a significant increase in economic and social welfare throughout the vast Asia and Australasia region, coastal and urban areas, and most urban ASEAN countries over the past few decades (1). This is evident from the upward real GDP growth trends, continuous improvement of living standards, and the average citizen's purchasing power (2). Thus, public and private healthcare funding has expanded because of socioeconomic progress. Most countries have strengthened their ability to invest in national networks of hospitals and primary care units to achieve the goal of universal health coverage, which is apparent from the impressive medical and health expenditures (3). This trend is also visible from Mongolia to South Korea and from Thailand to Malaysia (4).

While sustainable development goals have been achieved in healthcare in the vast Asian and Australasia region, there is a large array of significant challenges and obstacles (5) including population aging, innovation in medical technologies, and increased societal demand for them. In some countries, the growing gap in socioeconomic inequalities remains, given their large population as well as disparities in citizen income (6). These issues are reflected in heavy out-of-pocket expenditure, particularly for most Western Pacific regions except Japan (7). Severe costs of medical care, especially for expensive NCDs such as cancer or autoimmune diseases, have frequently caused catastrophic household expenditure for health care (8). Based on the major findings in the field of life-cycle theory (9), it is believed that consumption is much more closely tied to income than the life-cycle theory predicts (10). Therefore, patients in Western Pacific regions who will meet heavy out-of-pocket expenditure can do better by accumulating savings; however, utilizing their savings for better health coverage is still a challenge.

Additionally, there are other prominent bottleneck issues for the provision of accessible and equitable medical care in remote and rural areas. China has adopted superb insurance expansion strategies to eliminate these problems, with substantial success rates (11). Some middle-income ASEAN health systems still depend on foreign donor aid and development assistance for healthcare. The COVID-19 pandemic has created additional constraints, with massive losses of governmental budgetary revenue streams derived from tourism observed for several global tourist destinations such as Thailand, Singapore, and Indonesia (12). With ambitious development of the network of national health technology assessment agencies (INAHT), many resource constrains were partially addressed. South Korea remains at the forefront of policy development in the adoption of HTA, in decision making processes for marketing approvals, and in reimbursement of innovative pharmaceuticals.

This Research Topic attracted a total of ten contributions, of which eight were successfully published.

The first contribution is entitled “Measurement of Agricultural Green Development Level in the Three Province of Northeast China under the Background of Rural Vitalization Strategy.” This study presented an evaluation index system for agriculture green development level, which included four dimensions: agriculture development, ecological resource protection, environment-friendly, and industrial extension and integration. The study used the entropy-gray correlation method to calculate the overall development level, average variation speed, and four dimension levels in HeiLongjiang, Liaoning, and Jilin. Finally, the authors provided suggestions on how to promote the green development of agriculture-based calculation results (Hou and Wang).

The second contribution is entitled “Cost-Effectiveness of Posaconazole vs. First-Generation Triazoles for the Prevention of Invasive Fungal Infections Among High-Risk Patients With Hematological Malignancies in China.” The study built a hybrid decision tree and a Markov model to estimate and compare the cost-effectiveness of two antifungal prophylaxis regimens in hematological-malignancy Chinese patients at high risk of IFIS. The results demonstrated that a Posaconazole orla suspension provided an additional 0.109 QALYs at an incremental cost of $954.7, yielding an ICER of $8,784.4/QALY, which was below the national WTP threshold of $31,315/QALY. Scenario analyses showed the cost-effectiveness of Posaconazole ranged from 78.1% to 99.0%. Overall, Posaconazole was a highly cost-effective medicine for preventing IFI in high-risk hematological patients, and can be considered for use in some high-income regions of China (Shi et al.).

The third contribution is entitled “Simulating potential associated socio-economic determinants with sustainable food security (a new macro-micro spatial modeling approach).” The article assessed the geographical pattern of association between socioeconomic factors and food security in urban areas of Iran based on a nationally and regionally representative household consumption-expenditure survey during 2010–2018, which provided evidence of the relative merits. This study was the first attempt to assess the geographical differences of factors associated with food security by bringing together different studies from a variety of distinct disciplines. The study suggested more specific policies for each region to improve food security considering geographical disparities (Pakravan-Charvadeh et al.).

The fourth contribution is entitled “Who initiates prices competition when generic entrants are introduced into the South Korean pharmaceutical market?” The study measured the degree of price competition under different scenarios and utilized multilevel analysis to investigate the product and substance-level determinants. The results showed that the price competition was rare (only 11%) at the product level compared with that (43%) at the substance level, and the significant determinant was the maximum reimbursement price at the substance level in South Korea. The authors suggested the introduction of generic discounts to the health system (Son).

The fifth contribution is entitled “Impact of Mobile Payment on Physical Health: Evidence from the 2017 China Household Finance Survey.” The article used usual least squares and two-stage least squares strategies to explore the direct effects of mobile payment on physical health based on 2017 China Household Finance Survey data. The results showed that mobile payment had a positive impact on citizens' physical health, especially rural and less educated citizens, through the purchase of private health insurance and the cost of family entertainment (Zhang et al.).

The sixth contribution is entitled “Household Clean Energy Consumption and Health: Theoretical and Empirical Analysis.” The article examined the effect of the underline impact mechanism and its household energy consumption factors on residents' health, based on 2018 CHARLS data, using health economics theories, energy economics theories, and mediating effect models. The authors also studied the relationships between clean energy and chronic diseases and depression (Li et al.).

The seventh contribution is entitled “Patent foramen ovale closure vs. medical therapy alone after cryptogenic stroke in China: A cost-effectiveness analysis.” This was the first study to compare the effectiveness of PFO closure and medical therapy. The study established a 3-month Markov model and simulated 30-year QALY. The ICER of PFO was $3,783/QALY, which was lower than the ICER of medical therapy ($31,264/QALY) and WTP threshold ($47,654/QALY). Therefore, it was demonstrated that PFO closure was cost-effective for cryptogenic stroke patients with PFO in China (Wei et al.).

The eighth contribution is entitled “Research on the sustainable development of agricultural product supply chain in three northeast provinces in China.” The article used the entropy weight-matter element extension model and the autoregressive integrated moving average model to explore the sustainable development status from 2007 to 2020 and the trend in the next 5 years. The results can be applied to the governance of agricultural product supply chain in unevenly developed and remote regions (Fan et al.).

## Conclusive remarks

Global collaboration is the cornerstone of scientific and socioeconomic advancement. These contributions aim to shed light on the recent progress made across the entire breadth of the Public Health fields, Health Economics fields, and so on, and reflect on the future challenges faced by researchers in different countries (13).

We, the Editors, believe that these valuable and diverse Research Topic contributions may open a new horizon of knowledge. Last but not least, this is a unique opportunity to open the floor for a public debate on challenges in Asia (3). A heterogenous group of authors from academia, pharmaceutical and medical device industries, and governments have attempted to provide a thorough overview of the status of Health Economics in the Asian-Australasian economies (14). We hope that this article collection will trigger curiosity amongst aspiring Asian health economists and the lay audience alike (15).

## Author contributions

MJ and HC prepared the manuscript draft while MJ, HC, and NK revised it for important intellectual content. All authors contributed to the article and approved the submitted version.

## References

[1] Ozawa T. The rise of Asia. Books (2009).

[2] Jakovljevic M Timofeyev Y Ranabhat CL Fernandes PO Teixeira JP Rancic N. Real GDP growth rates and healthcare spending–comparison between the G7 and the EM7 countries. Global Health. (2020) 16:1–13. 10.1186/s12992-020-00590-3 32677998 PMC7367257

[3] Jakovljevic M Liu Y Cerda A Simonyan M Correia T Mariita RM. The Global South political economy of health financing and spending landscape–history and presence. J Med Econ. (2021) 24:25–33. 10.1080/13696998.2021.2007691 34866543

[4] Shao M Jin H Tsai FS Jakovljevic M. How fast are the Asian countries progressing toward green economy? Implications for public health. Front Public Health. (2022) 9:2365. 10.3389/fpubh.2021.753338 35198528 PMC8858809

[5] Sapkota B Palaian S Shrestha S Ozaki A Mohamed Ibrahim MI Jakovljevic M. Gap analysis in manufacturing, innovation and marketing of medical devices in the Asia-Pacific region. Expert Rev Pharmacoecon Outcomes Res. (2022) 22:1043–50. 10.1080/14737167.2022.2086122 35658768

[6] Boruzs K Juhász A Nagy C Szabó Z Jakovljevic M Bíró K. High inequalities associated with socioeconomic deprivation in cardiovascular disease burden and antihypertensive medication in Hungary. Front. Pharmacol. (2018) 9:839. 10.3389/fphar.2018.00839 30123128 PMC6085562

[7] Jakovljevic M Lamnisos D Westerman R Chattu VK Cerda A. Future health spending forecast in leading emerging BRICS markets in 2030: health policy implications. Health Res Policy Syst. (2022) 20:1–14. 10.1186/s12961-022-00822-5 35183217 PMC8857747

[8] Jakovljevic M Jakab M Gerdtham U McDaid D Ogura S Varavikova E. Comparative financing analysis and political economy of noncommunicable diseases. J Med Econ. (2019) 22:722–7. 10.1080/13696998.2019.1600523 30913928

[9] Browning M Deaton A Irish M. A Profitable approach to labor supply and commodity demands over the life-cycle. Econometrica. (1985) 53:503–43. 10.2307/1911653

[10] Deaton A. Measuring and understanding behavior, welfare, and poverty. Am Econ Rev. (2016) 106:1221–43. 10.1257/aer.106.6.1221

[11] Mallapaty S. China's five-year plan focuses on scientific self-reliance. Nature. (2021) 591:353–353. 10.1038/d41586-021-00638-3 33707693

[12] Chattu VK Singh B Kaur J Jakovljevic M. COVID-19 vaccine, TRIPS, and global health diplomacy: India's role at the WTO platform. Biomed Res Int. (2021) 2021:55. 10.1155/2021/6658070 34485525 PMC8416375

[13] Jakovljevic M Sugahara T Timofeyev Y Rancic N. Predictors of (in) efficiencies of healthcare expenditure among the leading asian economies–comparison of OECD and non-OECD nations. Risk Manag Healthc Policy. (2020) 13:2261. 10.2147/RMHP.S266386 33117004 PMC7585857

[14] Kim JI Lau LJ. The sources of Asian Pacific economic growth. Can J Econ. (1996) 29:S448–54. 10.2307/136085

[15] Jakovljevic M Wu W Merrick J Cerda A Varjacic M Sugahara T. Asian innovation in pharmaceutical and medical device industry–beyond tomorrow. J Med Econ. (2021) 24:42–50. 10.1080/13696998.2021.2013675 34915798

